# Type of facility influences lengths of stay of children presenting to high volume emergency departments

**DOI:** 10.1186/s12887-020-02400-6

**Published:** 2020-11-02

**Authors:** Rhonda J. Rosychuk, Brian H. Rowe

**Affiliations:** 1 Department of Pediatrics, Faculty of Medicine & Dentistry, University of Alberta, 3-524 Edmonton Clinic Health Academy, Edmonton, Alberta T6G 1C9 Canada; 2 Department of Mathematical and Statistical Sciences, University of Alberta, Edmonton, Alberta Canada; 3 Department of Statistics and Actuarial Science, Simon Fraser University, Burnaby, British Columbia Canada; 4 Department of Emergency Medicine, Faculty of Medicine & Dentistry, University of Alberta, Edmonton, Alberta T6G 2R7 Canada; 5 School of Public Health, University of Alberta, Edmonton, Alberta Canada

**Keywords:** Emergency department, Time to physician initial assessment, Length of stay, Wait times, Administrative data

## Abstract

**Background:**

Emergency department crowding may impact patient and provider outcomes. We describe emergency department crowding metrics based on presentations by children to different categories of high volume emergency departments in Alberta, Canada.

**Methods:**

This population-based retrospective study extracted all presentations made by children (age < 18 years) during April 2010 to March 2015 to 15 high volume emergency departments: five regional, eight urban, and two academic/teaching. Time to physician initial assessment, and length of stay for discharges and admissions were calculated based on the start of presentation and emergency department facility. Multiple metrics, including the medians for hourly, facility-specific time to physician initial assessment and length of stay were obtained.

**Results:**

About half (51.2%) of the 1,124,119 presentations were made to the two academic/teaching emergency departments. Males presented more than females (53.6% vs 46.4%) and the median age was 5 years. Pediatric presentations to the three categories of emergency departments had mostly similar characteristics; however, urban and academic/teaching emergency departments had more severe triage scores and academic/teaching emergency departments had higher admissions. Across all emergency departments, the medians of the metrics for time to physician initial assessment, length of stay for discharges and for admission were 1h11min, 2h21min, and 6h29min, respectively. Generally, regional hospitals had shorter times than urban and academic/teaching hospitals.

**Conclusions:**

Pediatric presentations to high volume emergency departments in this province suggest similar delays to see providers; however, length of stay for discharges and admissions were shorter in regional emergency departments. Crowding is more common in urban and especially academic emergency departments and the impact of crowding on patient outcomes requires further study.

**Supplementary Information:**

**Supplementary information** accompanies this paper at 10.1186/s12887-020-02400-6.

## Background

Emergency department (ED) crowding is an increasingly common concern for health care systems in developed countries. Crowding is a condition where the demand for services exceeds the ability to provide timely and high quality care [1]. There are many reasons why crowding is an important problem, including but not limited to: delays in delivering time-sensitive therapies such as analgesics [2–5] and antibiotics, [4, 6–10] increased departures prior to completion of care, [11–13] worse patient outcomes [14–20] and provider/patient dissatisfaction [14, 21, 22]. Research using population-based administrative data conducted in Ontario demonstrated that crowded EDs resulted in increased admissions and deaths [18].

The role of ED crowding in the care of pediatric patients is complicated by the fact that few studies have been performed. Pediatric patients may be seen in mixed EDs – which see both adult and pediatric patients – and the number of dedicated pediatric EDs is limited and restricted to 14 urban/academic centres across Canada. A study conducted in 2017 found a strong association between markers of crowding and pre-defined adverse outcomes in a pediatric ED in Vancouver, British Columbia [23].

There is no single universally accepted ED crowding metric [24] and multiple metrics have been defined that summarize aspects related to how patients arrive in the ED, the processes of care in the ED, and the patient’s disposition from the ED. [25] The Canadian Association of Emergency Physicians (CAEP) has recommended benchmarks for ED performance: [26] time to placement in an ED bed, time to physician initial assessment (PIA), and length of stay (LOS) in the ED (for admitted and discharged patients).

Our objectives were to provide a comprehensive picture of ED care for pediatric patients in one large geographic region and examine differences in crowding metrics in unique categories of EDs.

## Methods

### Study design

A population-based administrative health database of patient encounters from April 1, 2010, to March 31, 2015, from the Canadian province of Alberta was used to create this retrospective cohort study. Our data extract included both children and adults although separate studies have been conducted on those two populations. The methods for this paper follow those described in detail in an earlier publication [27]. The Health Research Ethics Board of the University of Alberta approved this study (Pro00056282).

### Study setting and population

ED presentations from high volume EDs during the study period were extracted from the National Ambulatory Care Reporting System (NACRS) including Alberta patients aged < 18 years at the time of the presentation, At the time of the study, Alberta had a population of more than 4 million and residents are registered in the Alberta Health Care Insurance Plan (AHCIP) that provides medically necessary health care in a uniform single-payer health system. Few patients (< 1%) present to the ED who are not registered in the AHCIP [28] and these patients were excluded.

The study focused on Alberta’s 15 highest volume EDs that care for patients in the relevant age group. The two academic/teaching EDs exclusively see pediatric patients (media*n* = 54,129 presentations/year), while all other EDs (*n* = 5 regional, *n* = 8 urban) assess a mixed patient population (regional: median = 57,307 presentations/year; urban: median = 54,502 presentations/year). Patients and the public were not involved in this research.

### Study protocol

The NACRS database contains demographic and geographic data collected at the time of the ED presentation including age in years and sex (male, female, or other, since other gender details were not collected). Place of residence at the time of presentation is reported in one of five health zones (North, Edmonton, Central, Calgary, South).

Dates and times related to an ED presentation, triage level, and disposition status are provided in the NACRS database. The date/time variables included the key times of process flow through the ED: registration, triage, physician initial assessment, disposition decision, and departure. The start of the ED presentation was set to be the minimum of the registration and triage dates and times and defined fiscal year, month of year, weekday/weekend, and time of shift (daytime 08:01–16:00, evening 16:01–24:00, night 00:01–08:00). The triage codes based on the Canadian Emergency Department Triage and Acuity Scale (CTAS) [29, 30] reflect the urgency of ED care required from resuscitation (1), emergency (2), urgent (3), semi-urgent (4), to non-urgent (5). Patients are assigned a disposition when released from the ED, they receive one of 10 disposition codes that we have grouped as discharges, admissions, transfers, deaths, and left without completion of care (e.g., patients who left the ED against medical advice (LAMA) or who left without being seen (LWBS)).

### Key outcome measures

Time to PIA and LOS have been recommended as ED crowding metrics [26] and we calculated those metrics as well as percent LAMA and percent LWBS based on all presentations made by patients (aged < 18 years) to a facility for any condition. Time to PIA was the time from the start of the ED presentation to the first assessment by a physician, and excludes patients who left without completion of care. These EDs are staffed by full-time emergency physicians and do not routinely employ diversion or advanced care nurses (e.g., nurse practitioners) in direct patient care. In critical situations, physician assessment may occur before or simultaneously with registration/triage, and thus there were some negative PIAs that we set to zero [31]. For discharged patients, the ED LOS was calculated as the time from the start of ED presentation until the time of disposition decision [31]. For admitted patients, the ED LOS was calculated as the time from the start of ED presentation until the time the patient left the ED for hospital admission [31]. The proportion of patients presenting who had a disposition of LAMA was calculated to obtain the LAMA proportion and similar calculations were made for the proportion LWBS.

All ED presentations for patients aged < 18 years that started within the same date and hour (e.g. 08:00–08:59) were determined for each ED facility and hourly facility-specific means, medians, and 90th percentiles for PIA (labeled PIA-A, PIA-M, PIA-90, respectively) and LOS (labeled LOS-A, LOS-M, LOS-90, respectively) were calculated. The facility-specific proportion LAMA and proportion LWBS were used for presentations that started on the same date (i.e., day aggregated metrics).

### Data analysis

Counts (percentages), means (standard deviations [SDs]), and medians (interquartile ranges [IQR] represented as [25th percentile, 75th percentile]) summarize patient demographics and ED presentation characteristics. For the continuous ED crowding metrics, summaries are provided for the mean, median, and 90th percentile of the measure. Statistical analyses were conducted in R (Vienna, Austria; Version 3.5.1) [32].

## Results

**Fig. 1:**
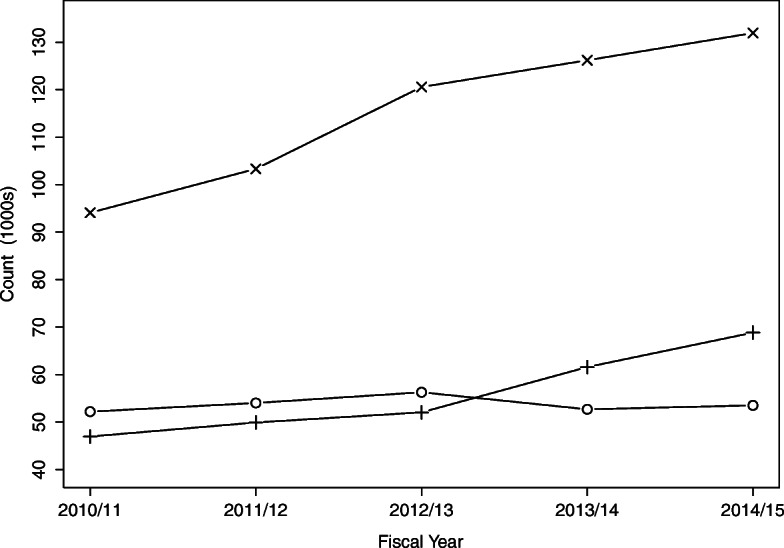
Emergency department presentations by fiscal year and ED category: regional (○), urban (+), and academic/teaching (×)

There were 465,709 children who made 1,124,119 presentations to these high volume EDs during the five year study period. Just over half (51.2%) of presentations were to the academic/teaching EDs that are dedicated pediatric EDs whereas regional EDs and urban EDs each had about 25% of the presentations (Table 1). Presentations were made by slightly more males than females (53.6% vs 46.4%), with a median age of 5.0 (IQR: 1, 12), and were made by children mainly in the most urbanized areas (Calgary and Edmonton). The number of presentations generally increased over time with the largest increases seen in the urban and academic/teaching EDs (Table 1, Fig. 1). Pediatric presentations to the ED were similar across ED categories for month, day of the week, and time. Urban and academic/teaching EDs had presentations with higher acuity than regional EDs (15.0, 12.8, and 7.7% were emergency triage level in the academic/teaching, urban, and regional EDs, respectively). Academic/teaching EDs had the highest proportion of admissions (7.8%) and smallest proportions of LWBS (2.8%) and LAMA (0.3%). Few presentations (*n* = 213) ended in death.

**Table 1:** Demographic and presentation characteristics of pediatric presentations to the 15 highest volume emergency departments in Alberta from 2010 to 2015 by departmental category

Characteristic	All EDs (*n*^a^ = 1,124,119)		Regional EDs (*n* = 268,683)		Urban EDs (*n* = 279,329)		Academic/ Teaching EDs (*n* = 576,107)	
Sex, *n* (%)								
Female	521,605	(46.4)	127,337	(47.4)	130,461	(46.7)	263,807	(45.8)
Male	602,511	(53.6)	141,346	(52.6)	148,868	(53.3)	312,297	(54.2)
Age (years)								
mean (SD^b^)	6.6	(5.7)	7.0	(5.9)	7.6	(6.0)	5.9	(5.5)
median [IQR^c^]	5.0	[1.0, 12.0]	5.0	[1.0, 13.0]	6.0	[2.0, 14.0]	4.0	[1.0, 10.0]
Zone of residence, *n* (%)								
Z5 North	141,728	(12.6)	128,050	(47.7)	3218	(1.2)	10,460	(1.8)
Z4 Edmonton	379,665	(33.8)	1604	(0.6)	175,953	(63.0)	202,108	(35.1)
Z3 Central	62,652	(5.6)	51,838	(19.3)	1640	(0.6)	9174	(1.6)
Z2 Calgary	442,157	(39.3)	2693	(1.0)	94,644	(33.9)	344,820	(59.9)
Z1 South	81,421	(7.2)	78,794	(29.3)	504	(0.2)	2123	(0.4)
Missing	16,496	(1.5)	5704	(2.1)	3370	(1.2)	7422	(1.3)
Fiscal year, *n* (%)								
2010/2011	193,231	(17.2)	52,191	(19.4)	46,956	(16.8)	94,084	(16.3)
2011/2012	207,257	(18.4)	54,019	(20.1)	49,912	(17.9)	103,326	(17.9)
2012/2013	228,885	(20.4)	56,272	(20.9)	52,045	(18.6)	120,568	(20.9)
2013/2014	240,467	(21.4)	52,686	(19.6)	61,582	(22.0)	126,199	(21.9)
2014/2015	254,279	(22.6)	53,515	(19.9)	68,834	(24.6)	131,930	(22.9)
Month of year, *n* (%)								
January	96,584	(8.6)	22,450	(8.4)	22,938	(8.2)	51,196	(8.9)
February	96,089	(8.5)	23,202	(8.6)	23,599	(8.4)	49,288	(8.6)
March	104,653	(9.3)	25,370	(9.4)	25,546	(9.1)	53,737	(9.3)
April	93,261	(8.3)	23,158	(8.6)	22,537	(8.1)	47,566	(8.3)
May	100,886	(9.0)	24,338	(9.1)	26,026	(9.3)	50,522	(8.8)
June	93,162	(8.3)	22,163	(8.2)	24,171	(8.7)	46,828	(8.1)
July	83,106	(7.4)	19,966	(7.4)	21,024	(7.5)	42,116	(7.3)
August	80,270	(7.1)	19,241	(7.2)	20,063	(7.2)	40,966	(7.1)
September	90,448	(8.0)	21,488	(8.0)	23,166	(8.3)	45,794	(7.9)
October	92,384	(8.2)	21,893	(8.1)	23,095	(8.3)	47,396	(8.2)
November	91,971	(8.2)	21,773	(8.1)	22,568	(8.1)	47,630	(8.3)
December	101,305	(9.0)	23,641	(8.8)	24,596	(8.8)	53,068	(9.2)
Day of week, n (%)								
Weekday (Mon-Fri)	783,905	(69.7)	185,264	(69.0)	192,291	(68.8)	406,350	(70.5)
Weekend (Sat, Sun)	340,214	(30.3)	83,419	(31.0)	87,038	(31.2)	169,757	(29.5)
Time of day, *n* (%)								
Daytime (08:01–16:00)	449,174	(40.0)	110,186	(41.0)	99,088	(35.5)	239,900	(41.6)
Evening (16:01–24:00)	531,261	(47.3)	127,687	(47.5)	141,119	(50.5)	262,455	(45.6)
Night (00:01–08:00)	143,684	(12.8)	30,810	(11.5)	39,122	(14.0)	73,752	(12.8)
Triage level, *n* (%)								
1 Resuscitation	4228	(0.4)	472	(0.2)	755	(0.3)	3001	(0.5)
2 Emergency	142,700	(12.7)	20,669	(7.7)	35,636	(12.8)	86,395	(15.0)
3 Urgent	511,712	(45.5)	104,049	(38.7)	131,375	(47.0)	276,288	(48.0)
4 Semi-urgent	412,229	(36.7)	130,364	(48.5)	91,550	(32.8)	190,315	(33.0)
5 Non-urgent	52,460	(4.7)	12,419	(4.6)	19,989	(7.2)	20,052	(3.5)
Missing	790	(0.1)	710	(0.3)	24	(0.0)	56	(0.0)
Disposition, *n* (%)								
Discharged	997,047	(88.7)	235,917	(87.8)	252,051	(90.2)	509,079	(88.4)
Admitted	63,695	(5.7)	15,920	(5.9)	2735	(1.0)	45,040	(7.8)
Transferred	11,363	(1.0)	1013	(0.4)	6462	(2.3)	3888	(0.7)
LWBS^d^	45,833	(4.1)	14,026	(5.2)	15,833	(5.7)	15,974	(2.8)
LAMA^e^	5968	(0.5)	1748	(0.7)	2204	(0.8)	2016	(0.3)
Death	213	(0.0)	59	(0.0)	44	(0.0)	110	(0.0)

^a^ *n* count, ^b^ *SD* standard deviation, ^c^*IQR* 25th percentile, 75th percentile;

^d^ *LWBS* left without being seen;

^e^ *LAMA* left against medical advice

Two urban EDs opened at 7 am on January 14, 2013, and May 21, 2014, and the remaining 13 EDs were operational for the full duration of the five-year study period. Thus, a total of 596,628 facility-specific hours were used in the calculation of metrics during the study period. Some facilities and hours had missing values because there may have been no children presenting or presentations with non-missing times did not meet the disposition requirements (e.g., PIA based on 304,959 facility-specific hours, ED LOS for discharged based on 62,033 facility-specific hours, and ED LOS for admitted based on 37,574 facility-specific hours).

**Fig. 2:**
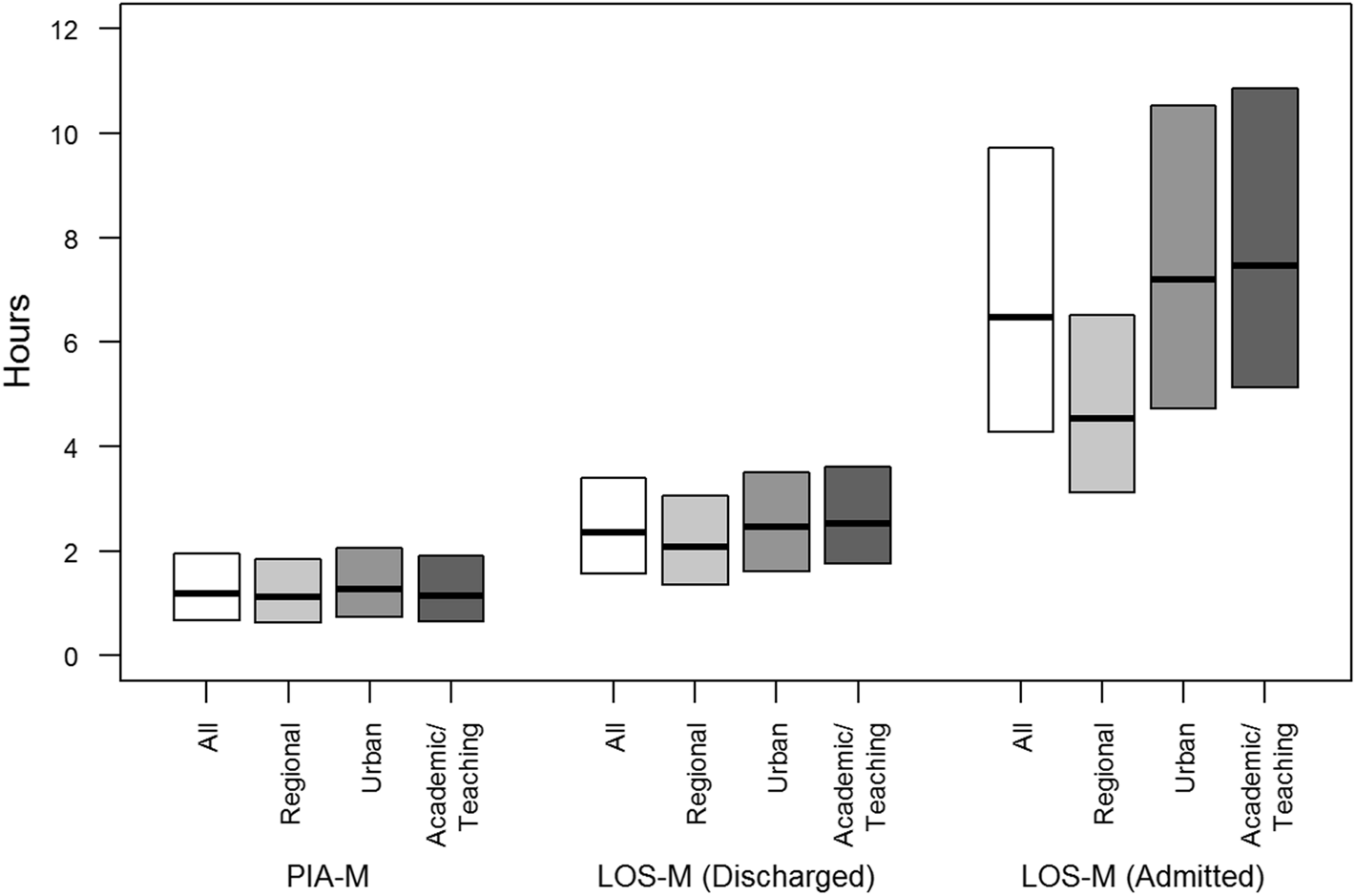
Median and interquartile range (25th percentile, 75th percentile) for hourly, facility-specific median physician initial assessment (PIA-M) times, median length of stay (LOS-M) for discharged patients, and median length of stay (LOS-M) for admitted patients for all EDs and by ED category

The metrics based on PIA were similar across the ED categories (Table 2, Fig. 2). The median of the PIA-M metric was 1h7min (IQR 37 min, 1h51min) for the regional EDs, 1h15min (IQR 43 min, 2h2min) for the urban EDs, and 1h8min (IQR 39 min, 1h54min) for the academic/teaching EDs. The PIA-M metric remained relatively stable over time (Additional file 1 Fig. 1). Of 916,641 ED visits where PIA could be calculated, 517,150 (56.4%) exceeded 1 h and 87,952 (9.6%) exceeded 3 h.

**Table 2:** Summaries of hourly, facility-specific crowding metrics for all EDs and by ED category

Crowding Metric	All EDs		Regional EDs		Urban EDs		Academic/Teaching EDs	
PIA-A^a^								
median [IQR^b^]	1h12min	[42 min, 1h56min]	1h08min	[38 min, 1h50min]	1h16min	[44 min, 2h02min]	1h11min	[42 min, 1h53min]
PIA-M^c^								
median [IQR]	1h11min	[40 min, 1h57min]	1h07min	[37 min, 1h51min]	1h15min	[43 min, 2h02min]	1h08min	[39 min, 1h54min]
PIA-90^d^								
median [IQR]	1h25min	[48 min, 2h20min]	1h16min	[41 min, 2h04min]	1h27min	[48 min, 2h22min]	1h35min	[54 min, 2h33min]
LOS-A^e^ for discharged patients								
median [IQR]	2h27min	[1h38min, 3 h29min]	2h08min	[1h23min, 3 h05min]	2h29min	[1h39min, 3 h32min]	2h45min	[1h58min, 3 h49min]
LOS-M^f^ for discharged patients								
median [IQR]	2h21min	[1h34min, 3 h24min]	2h05min	[1h21min, 3 h03min]	2h27min	[1h37min, 3 h31min]	2h31min	[1h45min, 3 h37min]
LOS-90^g^ for discharged patients								
median [IQR]	2h54min	[1h50min, 4h13min]	2h25min	[1h31min, 3 h32min]	2h46min	[1h47min, 4h01min]	3h46min	[2h34min, 5h11min]
LOS-A for admitted patients								
median [IQR]	6h31min	[4h17min, 9h46min]	4h32min	[3h07min, 6h31min]	7h12min	[4h44min, 10h32min]	7h31min	[5h09min, 10h54min]
LOS-M for admitted patients								
median [IQR]	6h29min	[4h16min, 9h43min]	4h32min	[3h07min, 6h31min]	7h12min	[4h44min, 10h32min]	7h28min	[5h07min, 10h51min]
LOS-90 for admitted patients								
median [IQR]	6h45min	[4h22min, 10h16min]	4h35min	[3h08min, 6h36min]	7h12min	[4h45min, 10h34min]	7h54min	[5h22min, 11h37min]
Percent LWBS^h^ (Day aggregated)								
median [IQR]	2.8	[0.0,7.7]	3.7	[0.0,7.7]	4.0	[0.0,9.1]	0.0	[0.0,4.1]
Percent LAMA^i^ (Day aggregated)								
median [IQR]	0.0	[0.0,0.0]	0.0	[0.0,0.0]	0.0	[0.0,0.0]	0.0	[0.0,0.0]

^a^ *PIA-A* hourly, facility specific average time to physician initial assessment;

^b^ *IQR* 25th percentile, 75th percentile;

^c^ *PIA-M* hourly, facility specific median time to physician initial assessment;

^d^ *PIA-90* hourly, facility specific 90th percentile of time to physician initial assessment;

^e^ *LOS-A* hourly, facility specific average length of stay;

^f^ *LOS-M* hourly, facility specific median length of stay;

^g^ *LOS-90* hourly, facility specific 90th percentile of length of stay;

^h^ *LWBS* left without being seen;

^i^ *LAMA* left against medical advice

For presentations ending in discharge, the median LOS-M metric was 2h21min (IQR 1h34min, 3 h24min). The median LOS-M metric was highest for presentations at academic/teaching EDs (2h31min) and urban EDs (2h27min), and was lowest for regional EDs (2h05min). The median LOS-M metric for discharges remained relatively stable over time (Additional file 1 Fig. 2). The summary statistics for the LOS-A metric were close to the summary statistics for the LOS-M metric. When the 90th percentile is examined, the ED categories differed with median LOS-90 metrics of 3 h46min for the academic/teaching EDs, 2h46min for the urban EDs, and 2h25min for the regional EDs. Of the 996,380 discharges with times and triage level, 185,983 (18.7%) had LOS greater than 4 h (24.2% exceeded 4 h for CTAS 1, 2, or 3; 11.9% exceeded 4 h for CTAS 4 or 5 recommendations [26]).

Presentations ending in admission had longer LOSs than presentations ending in discharge: the median of the LOS-M metric was equal to 6h29min (IQR 4h16min, 9h43min). Academic/teaching EDs had the highest median of the LOS-M metric (7h28min), followed by urban (7h12min) and regional (4h32min) EDs. These metrics generally increased in the most recent years with more variability seen for the urban EDs (Additional file 1 Fig. 3). The summary statistics for the LOS-A metric were very close to the summary statistics for the LOS-M metric. When the 90th percentile was examined, the ED categories differed with median of the LOS-90 metric equal to of 7h54min for the academic/teaching EDs, 7h12min for the urban EDs, and 4h35min for the regional EDs. Of the 63,695 admissions with non-missing times, 23,380 (36.7%) had LOS greater than 8 h recommended [26] as the median LOS for admitted patients.

For the day aggregated metric of LWBS, the median percent LWBS was 0, 4.0, and 3.7% in academic/teaching, urban, and regional EDs, respectively (Table 2, Additional file 1 Fig. 4). Over time, LWBS has been variable with increases seen in more recent years for the academic/teaching EDs (Additional file 1 Fig. 5). Very few presentations ended with LAMA and the median percent LAMA was 0 across all categories of EDs and the mean percent LAMA was 1.2, 0.7, and 0.6% in academic/teaching, urban, and regional EDs, respectively.

## Discussion

This study focused on five years of population-based administrative data on presentations made by children (age < 18 years) to 15 high volume EDs in one Canadian province. High volume EDs are the most likely EDs to experience crowding and we described the crowding metrics PIA, LOS for discharges, LOS for admissions, LWBS, and LAMA that were obtainable from administrative data sources. These metrics focus on the efficiency (i.e., PIA, LOS) and safety (i.e., LWBS, LAMA) domains of medical care [33] and throughput (i.e., PIA, LOS) and output (i.e., LWBS, LAMA) aspects of the conceptual model of ED crowding [25]. Across three hospital categories, the patient demographics were similar: however, the academic/teaching EDs had the highest acuity scores and the highest proportion of admissions. Regional EDs had the lowest acuity scores and the lowest proportion of admissions.

The ED volumes for children have increased substantially over time for the academic/teaching and urban EDs yielding 40 and 47% increases during the 2010/2011 to 2014/2015 study period, respectively. These increases exceeded the 10% increase in the population of children and youth (≤19 years) [34] and the 12% increase in ED presentations for all ages, and all ED facilities during the same period [35, 36]. The disproportionate increases to the academic/teaching and urban EDs may reflect preferential choices for care in urban centres or lack of access to other care options. Urban EDs in the United States have seen an estimated increase in ED presentations during 2005 to 2016 of about 19% for all ages [37] and other jurisdictions have seen increases in ED presentations, [38, 39] many above the rate of population growth [38].

The crowding metrics based on delays in initial assessment were similar across ED category and time. The CAEP benchmark for PIA is a median of 1 h, [26] and 56% of the ED visits exceeded 1 h and the median of the PIA-Ms by hour and ED facility exceeded that recommendation by 11 min. Those results are similar to other Canadian studies where at Ottawa, Ontario’s children’s hospital during four, 2-week study periods during 2011/2012 where the median PIA was 1h30min, [40] in Ontario EDs during 2012 the median PIA as approximately 1 h, [39] and in urban EDs in Vancouver, British Columbia during 2012 the median PIA was approximately 1h10min [39]. In a Columbus, Ohio children’s hospital during 2016/17 the estimated mean PIA was 1h34min [41]. In a Cincinnati, Ohio pediatric teaching hospital during 2003 to 2007, the daily mean PIA was 53.6 m and increases in ED census was associated with increased PIA [42]. Crowding may increase PIA and such increases will increase the ED LOS.

Our study showed that the LOS metrics for both discharged and admitted patients was shortest for regional EDs. Academic/teaching EDs had the longest LOS metrics for both discharged patients (median of LOS-M = 2h31min) and admitted patients (median of LOS-M = 7h28min). The LOS for admitted patients has increased over recent years, bed capacity is low, regionalization of pediatric beds has occurred, and increased demands for inpatient beds for ED patients results in delays from a decision to admit to the time when an inpatient bed is available (so called “access block”). The LOS metrics for both discharged and admitted patients in this study are larger than EDs in Ontario (median LOS just under 2 h), [39] urban EDs in Vancouver (median LOS just under 2h30min), [39] and a Cincinnati pediatric teaching hospital (daily mean LOS was 2h30min) [42]. Another Ohio children’s hospital during 2016/17 had an estimated mean LOS of 4h10min [41] and in the United States national, the estimated median LOS for discharged patients of any age was 2h19min and for admitted patients was 4h22min in 2013 [43]. While the LOS metrics in our study were longer than in other studies, most of the ED presentations met the recommendations for LOS [26]. The longer LOS metrics in academic/teaching EDs compared to regional EDs may be directly related to the higher acuity and admission rates in academic/teaching EDs. Longer LOS in urban and academic/teaching EDs were not primarily a result of increased PIA as the PIA was similar across EDs.

The proportion of LWBS was small (all EDs: 4.1%) and variable, ranging from 2.8% for academic/teaching EDs to 5.7% for urban EDs. These results were similar to other jurisdictions in Canada. In Ontario EDs in 2008, 4.9% of presentations by children ended in LWBS and in urban Vancouver EDs LWBS occurred in 3–4% of presentations [39]. That same study found that LWBS did not change substantially with increasing volumes or acuity, and some centres in both provinces have participated in strategies to reduce ED wait times [39]. In our study, very few patients left against medical advice (all EDs: 0.5%), ranging from 0.3% for academic/teaching EDs to 0.8% for urban EDs. This means that while the average daily LAMA was 1.2% for academic/teaching EDs, it was only 0.6 and 0.7% for the regional and urban EDs, respectively. There was more variability in the daily metrics for academic/teaching EDs in our study that may be because of higher volumes and more variability in crowding during different times of day.

The implications of this study vary based on one’s perspective. For example, for patients and families, emergency pediatric care is a priority and efforts to ensure government ministries are aware and responsive should be a priority. Alternatively, delays in urban Pediatric EDs are impressive and implementing throughput interventions (e.g., fast track, staffing models, rapid assessment zones, etc.) require urgent attention. For the health care system, increasing the availability of primary care providers (e.g., Family Physicians and Pediatricians) and access to same or next day primary pediatric care may reduce ED visits in the larger centres. Clearly, each zone and hospital is unique and solutions need to be based on an understanding of the bottlenecks and evaluated to determine effectiveness.

### Limitations

This study has both strengths and weaknesses. Strengths of our study include a large sample size from population-based data source in a geographically large area of Canada.. Compared to some of the other studies, this study focused on more than one ED, covered a large time period, included both pediatric and mixed age EDs, and examined the type of ED. As a limitation, our results may not be generalizable to other areas of Canada or other jurisdictions with different health care systems. The ED crowding metrics were not tested against important patient outcomes (e.g., in-patient LOS, re-visits to ED, death), and we cannot conclude any consequences regarding the degree of overcrowding observed. Finally, there may be errors in the documented times and some times were not available.

## Conclusions

This robust and comprehensive five-year study demonstrates that ED presentations for children have increased over time across the province, especially in the urban and academic/teaching hospitals. Metrics for PIA were similar across ED category whereas LOS for discharges and admissions varied by ED category. Crowding is more common in urban and academic/teaching EDs. The impact of crowding on outcomes for children presenting to the ED requires further study, especially in crowded academic/teaching hospitals. Moreover, interventions to address ED crowing in these locations are urgently required.

## Supplementary Information

**Additional file 1.** Figure 1. Median and interquartile range (25th percentile, 75th percentile) for hourly, facility-specific median physician initial assessment (PIA-M) times by years and by ED category. Figure 2. Median and interquartile range (25th percentile, 75th percentile) for hourly, facility-specific median length of stay (LOS-M) for discharges by years and by ED category. Figure 3. Median and interquartile range (25th percentile, 75th percentile) for hourly, facility-specific median length of stay (LOS-M) for admissions by years and by ED category. Figure 4. Median and interquartile range (25th percentile, 75th percentile) of daily, facility-specific percent left without being seen (LWBS) and left against medical advice (LAMA) for all EDs and by ED category. Figure 5. Median and interquartile range (25th percentile, 75th percentile) for daily, facility-specific percent left without being seen (LWBS) by years and by ED category.

## Data Availability

Data is the property of Alberta Health and the authors are not allowed to provide the data. Requests can be made for the same data from Alberta Health for researchers who meet the criteria for access to confidential data. Researchers are welcome to inquire for further information at Health.RESDATA@gov.ab.ca.

## Abbreviations

AHCIP: Alberta Health Care Insurance Plan
CTAS: Canadian Triage and Acuity Score
ED: Emergency department
h: Hours
IQR: Interquartile range represented as 25th percentile, 75th percentile
LAMA: Left against medical advice
LOS: Length of stay
LOS-A: Hourly facility-specific mean of LOS
LOS-M: Hourly facility-specific median of LOS
LOS-90: Hourly facility-specific 90th percentile of LOS
LWBS: Left without being seen
min: Minutes
n: Count
NACRS: National Ambulatory Care Reporting System
PIA: Time to physician initial assessment
PIA-A: Hourly facility-specific mean of PIA
PIA-M: Hourly facility-specific median of PIA
PIA-90: Hourly facility-specific 90th percentile of PIA
SD: Standard deviation
