# Supplementary material for: Type of facility influences lengths of stay of children presenting to high volume emergency departments
Source: BMC Pediatr. 2020 Nov 2;20:500. doi: 10.1186/s12887-020-02400-6 (PMC7604957; doi:10.1186/s12887-020-02400-6)

**Additional File 1**

Figure 1. Median and interquartile range (25th percentile, 75th percentile) for hourly, facility-specific median physician initial assessment (PIA-M) times by years and by ED category.


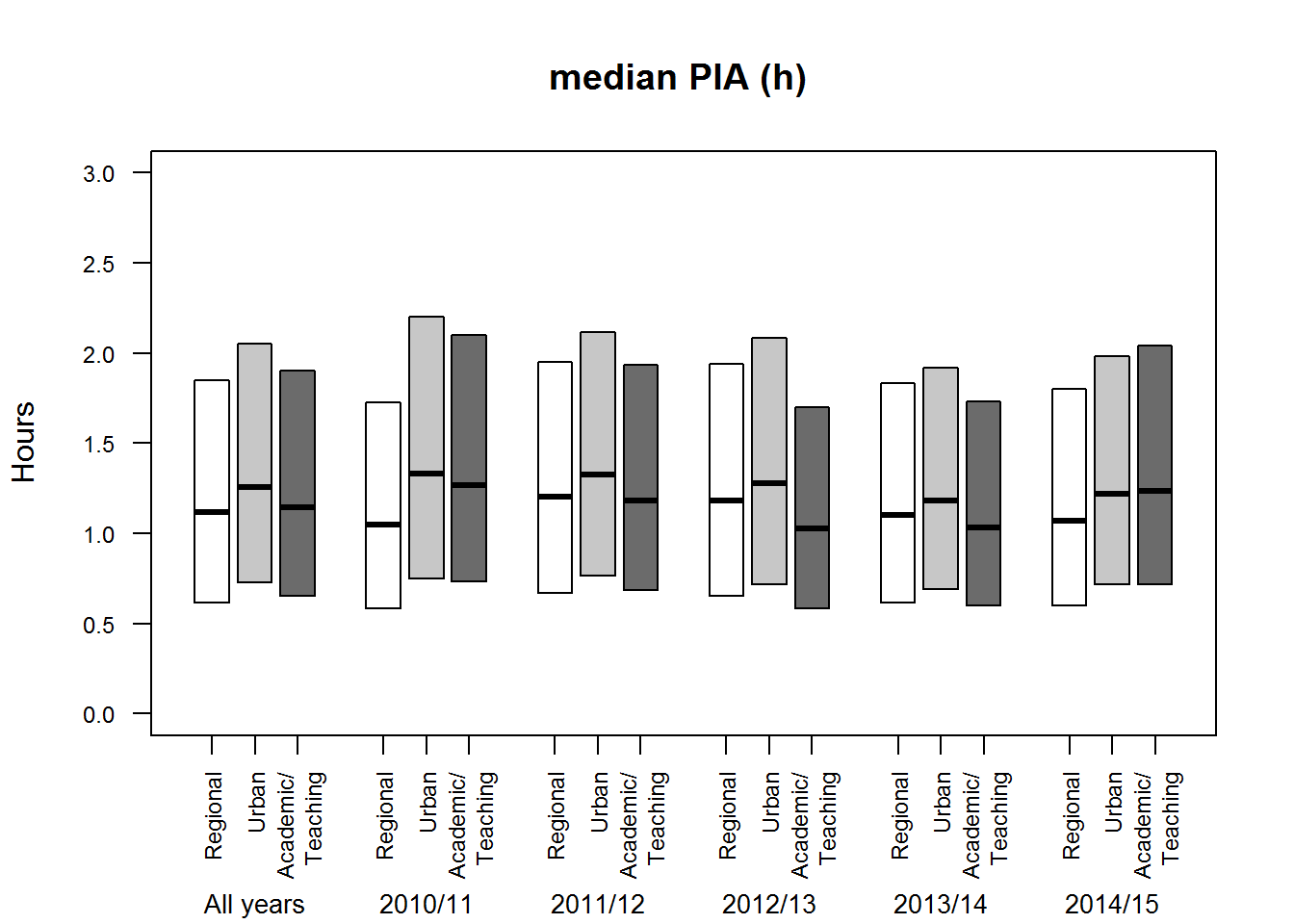


Figure 2. Median and interquartile range (25th percentile, 75th percentile) for hourly, facility-specific median length of stay (LOS-M) for discharges by years and by ED category.


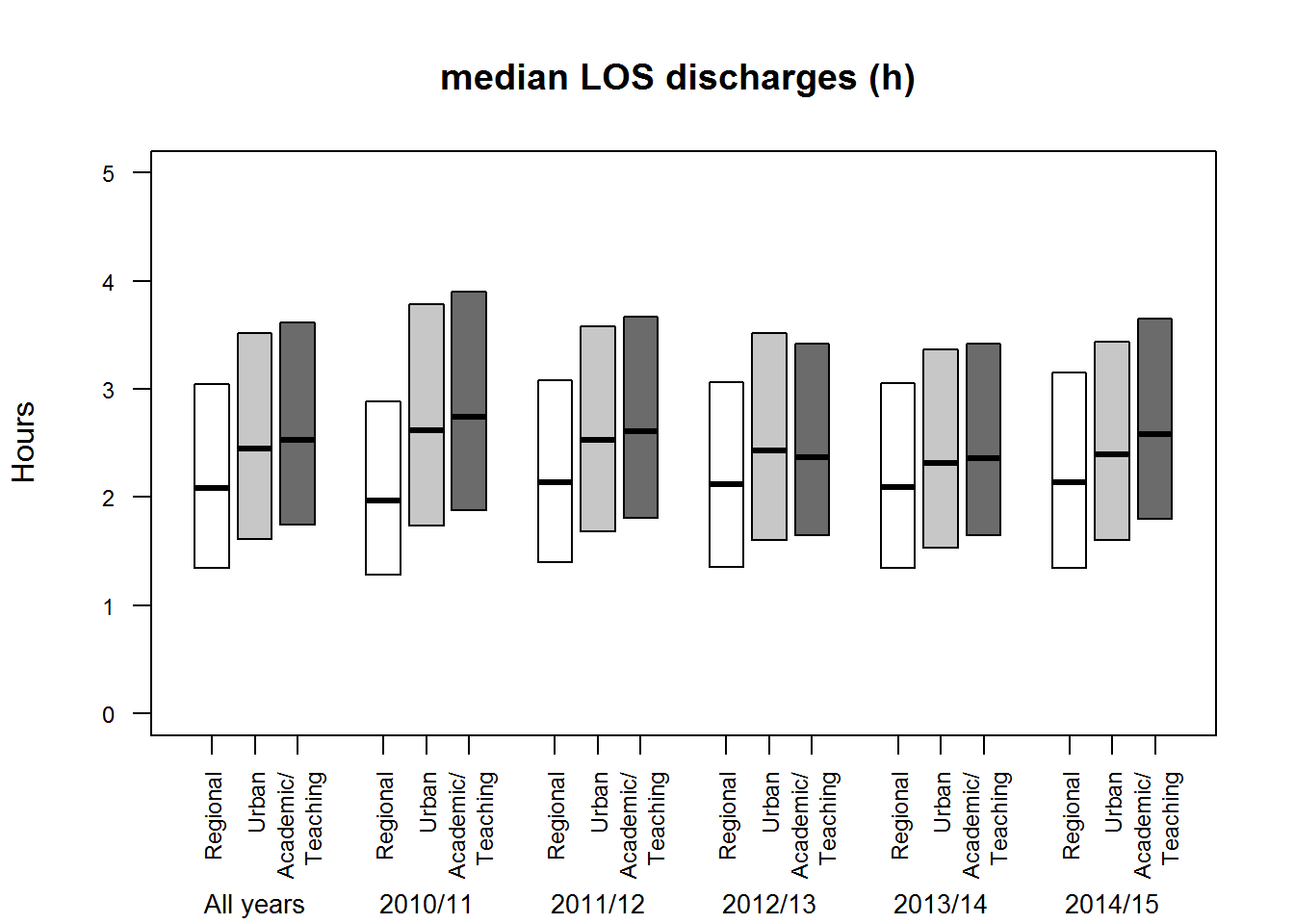


Figure 3. Median and interquartile range (25th percentile, 75th percentile) for hourly, facility-specific median length of stay (LOS-M) for admissions by years and by ED category.


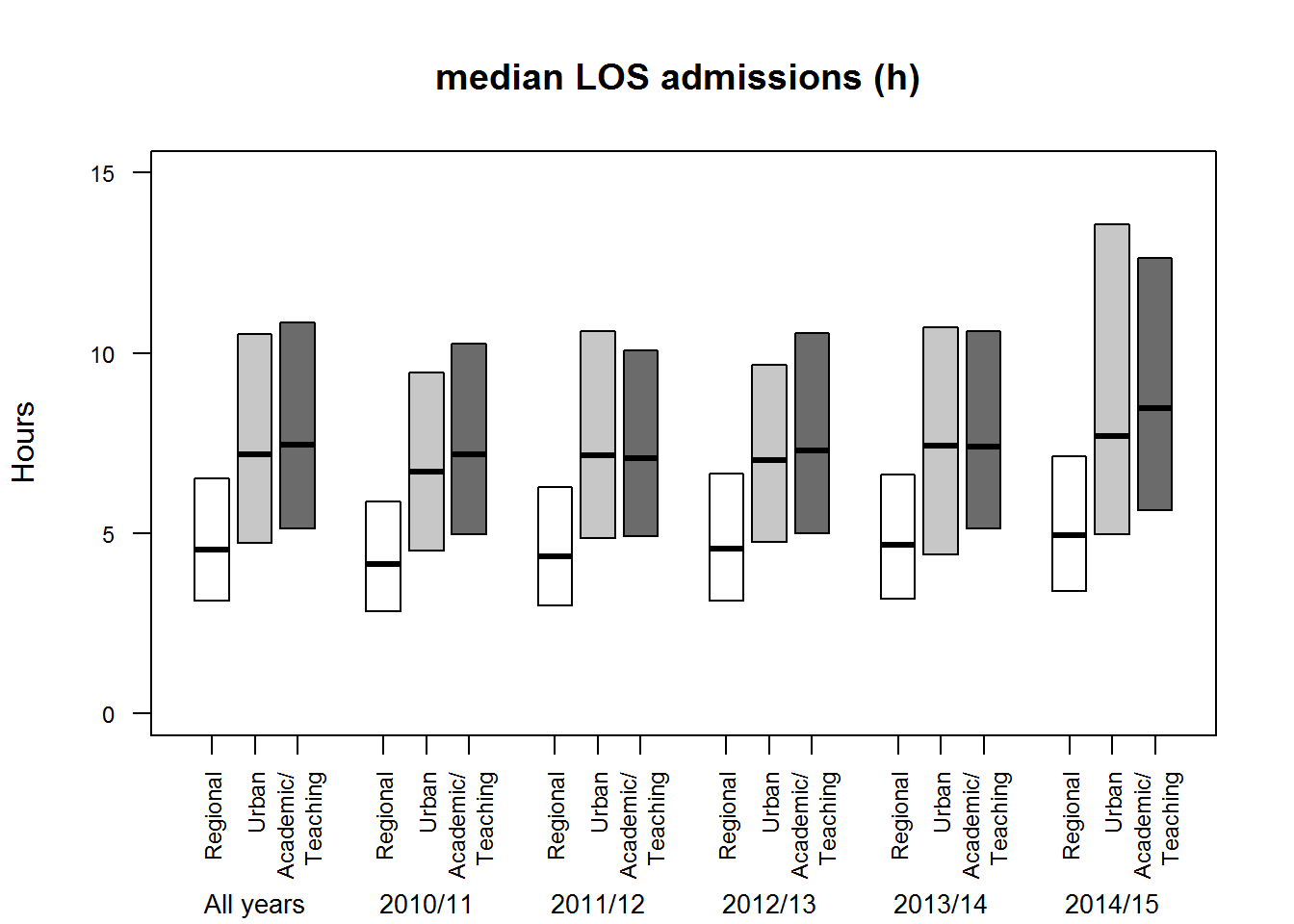


Figure 4. Median and interquartile range (25^th^ percentile, 75^th^ percentile) of daily, facility-specific percent left without being seen (LWBS) and left against medical advice (LAMA) for all EDs and by ED category.


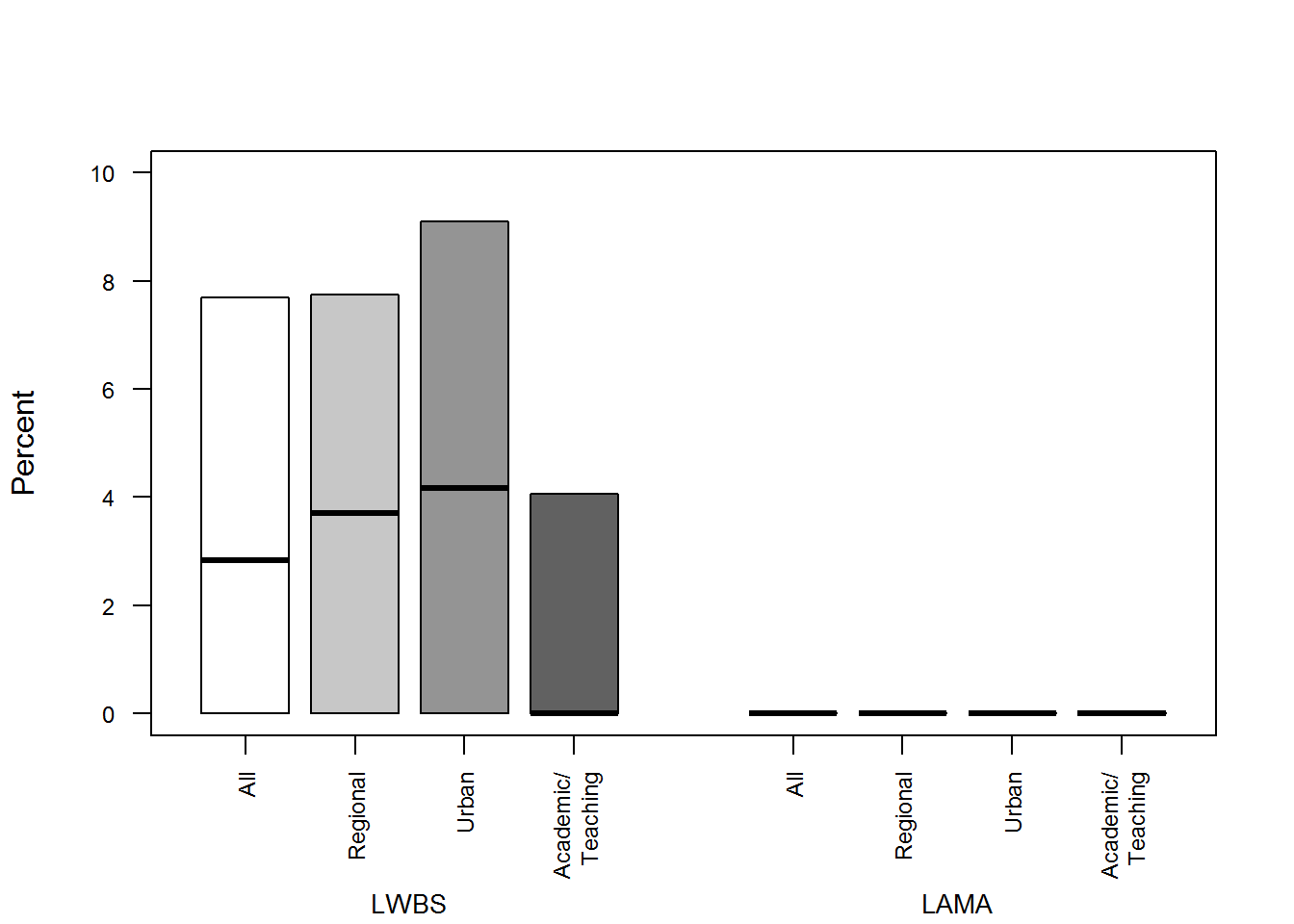


Figure 5. Median and interquartile range (25th percentile, 75th percentile) for daily, facility-specific percent left without being seen (LWBS) by years and by ED category.


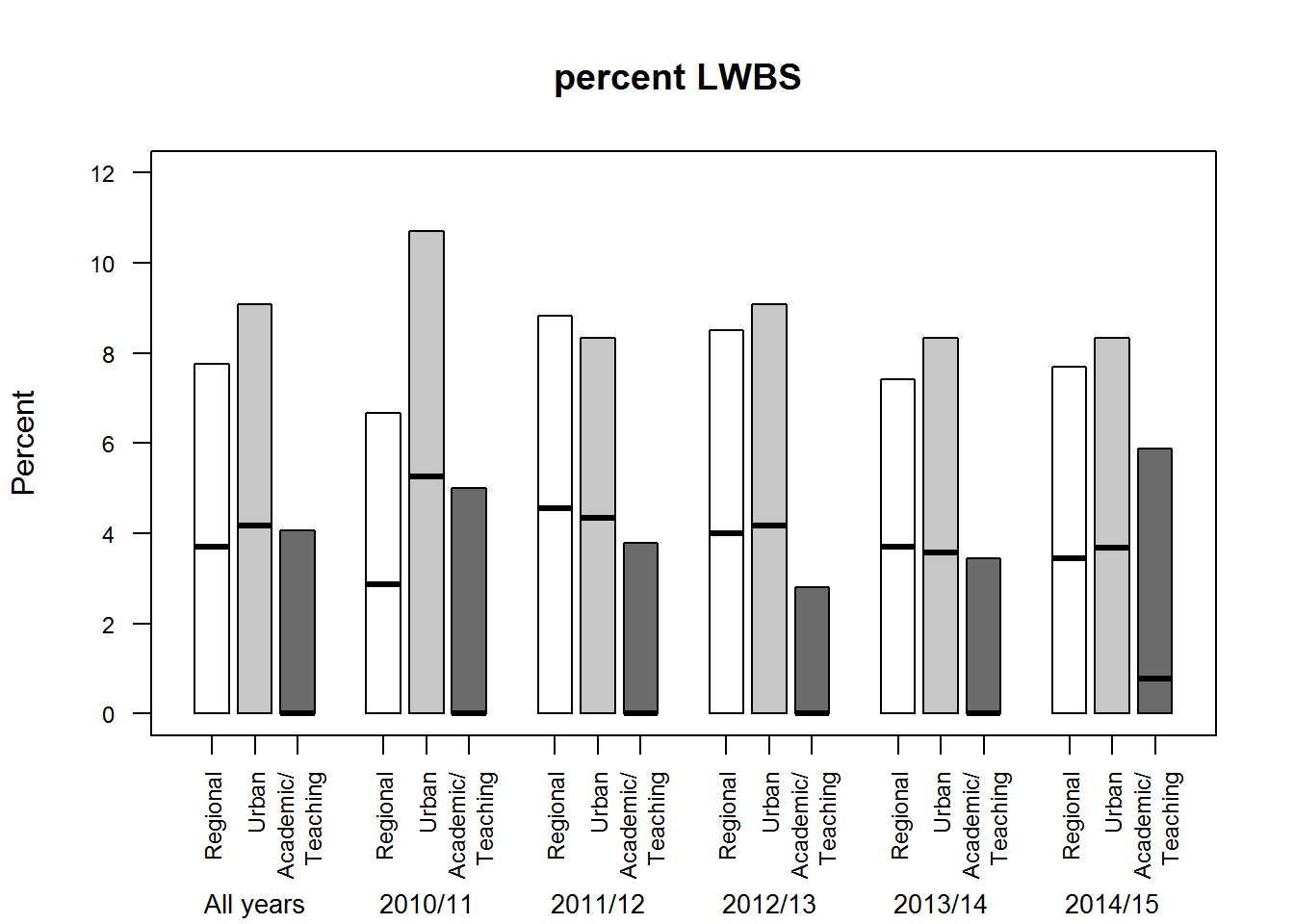

Supplement: Supplementary file 1 — Additional file 1. Figure 1. Median and interquartile range (25th percentile, 75th percentile) for hourly, facility-specific median physician initial assessment (PIA-M) times by years and by ED category. Figure 2. Median and interquartile range (25th percentile, 75th percentile) for hourly, facility-specific median length of stay (LOS-M) for discharges by years and by ED category. Figure 3. Median and interquartile range (25th percentile, 75th percentile) for hourly, facility-specific median length of stay (LOS-M) for admissions by years and by ED category. Figure 4. Median and interquartile range (25th percentile, 75th percentile) of daily, facility-specific percent left without being seen (LWBS) and left against medical advice (LAMA) for all EDs and by ED category. Figure 5. Median and interquartile range (25th percentile, 75th percentile) for daily, facility-specific percent left without being seen (LWBS) by years and by ED category. [file 12887_2020_2400_MOESM1_ESM.docx]
